# A case report on recurrent alternating Tolosa-Hunt syndrome due to bacterial sphenoid sinusitis: rediscussing the diagnostic terminology and classification

**DOI:** 10.1186/s12883-023-03067-z

**Published:** 2023-01-17

**Authors:** Wei He, Yinglin Zhu, Yinan Zhang, Liang Dong, Zefang Zhou, Jiying Zhou

**Affiliations:** 1 Department of Neurology, The First Branch of The First Affiliated Hospital of Chongqing Medical University, 191st Ren Min Road, Yu Zhong District, Chongqing, 400015 China; 2 School of Osteopathic Medicine, Kansas City University of Medicine and Biosciences, Joplin, MO 64801 USA; 3 Department of Neurology, The First Affiliated Hospital of Chongqing Medical University, 1st You Yi Road, Yu Zhong District, Chongqing, 400016 China

**Keywords:** Tolosa-Hunt syndrome (THS), Painful ophthalmoplegia, Cavernous sinus syndrome, Diagnostic criteria, ICHD-3

## Abstract

**Background:**

Tolosa-Hunt syndrome (THS) is characterized by painful ophthalmoplegia caused by idiopathic granulomatous inflammation involving the cavernous sinus region. Patients respond well to steroid therapy. THS is included in the differential diagnosis of cavernous sinus syndrome, so it is important to fully exclude other lesions in this area before treatment, otherwise steroid treatment may lead to fatal outcomes. Here we describe a patient who initially presented with symptoms that simulated THS symptoms and developed recurrent alternating painful ophthalmoplegia during follow-up, and the patient was finally diagnosed with cavernous sinusitis caused by bacterial sphenoid sinusitis.

**Case presentation:**

A 34-year-old woman presented with left painful ophthalmoplegia. Magnetic resonance imaging (MRI) revealed abnormal signals in the left cavernous sinus area, and these abnormal signals were suspected to be THS. After steroid treatment, the patient obtained pain relief and had complete recovery of her ophthalmoplegia. However, right painful ophthalmoplegia appeared during the follow-up period. MRI showed obvious inflammatory signals in the right cavernous sinus and right sphenoid sinus. Then nasal sinus puncture and aspiration culture were performed, and the results showed a coagulase-negative staphylococcus infection. After antibiotic treatment with vancomycin, the painful ophthalmoplegia completely resolved, and the neurological examination and MRI returned to normal.

**Conclusion:**

Some other causes of painful ophthalmoplegia also fulfill the diagnostic criteria for THS in the International Classification of Headache Disorders third edition (ICHD-3) and respond well to steroid therapy. Early diagnosis of THS may be harmful to patients, and clinicians should exercise great caution when dealing with similar cases without a biopsy. Using “cavernous sinus syndrome” instead of “Tolosa-Hunt syndrome” as a diagnostic category may provide a better clinical thinking for etiological diagnosis.

## Background

Tolosa-Hunt syndrome (THS) is characterized by unilateral orbital or periorbital pain accompanied by paralysis of cranial nerves III, IV, and/or VI [1, 2]. This ophthalmoplegia and pain are caused by a nonspecific aseptic inflammatory granuloma in the cavernous sinus, supraorbital fissure or orbital region [3]. THS is relatively rare clinically, and patients respond quickly to steroid therapy [4]. Since it is essentially a self-limiting disease, it is often considered benign. However, the clinical manifestations of THS are nonspecific and overlap with those of many other, potentially dangerous etiologies in the cavernous sinus region [5–8]. The third edition of the International Classification of Headache Disorders (ICHD-3) recommends close follow-up and repeat neuroimaging as appropriate to rule out other causes of painful ophthalmoplegia, such as tumor, vasculitis, skull base meningitis, sarcoidosis, or diabetes [9]. We report a case of alternating episodes of THS with a relapsing-remitting course. During the follow-up, a special clinical course and abnormal radiological changes were observed, and the lesion was eventually was diagnosed as cavernous sinus infection caused by bacterial sphenoid sinusitis.

## Case presentation

**Fig. 1:**
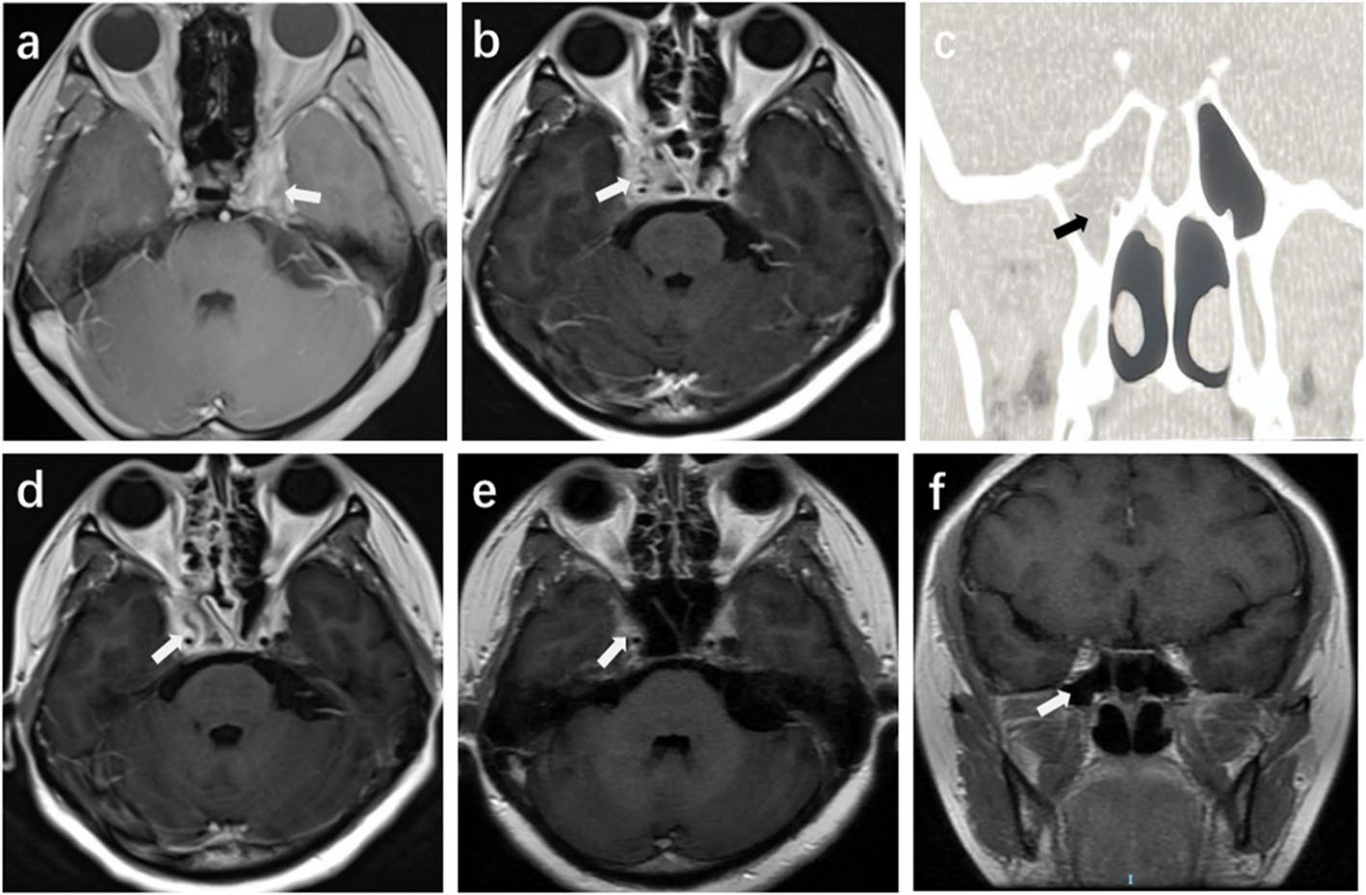
**a** Contrast-enhanced coronal T1 MRI of the first headache attack (left headache) on December 12, 2020. The left cavernous sinus was enlarged and widened. The abnormal tissue was strongly enhanced after the intravenous injection of the contrast material. **b** Contrast-enhanced coronal T1 MRI of the second headache attack (right headache) on January 10, 2021. The increased soft tissue shadows were present in the bilateral cavernous sinuses and were more pronounced on the right. **c** Axial sinus CT on January 14, 2021 shows inflammation of the right sphenoid sinus cavity. **d** One month after surgical and antibiotic therapy, MRI showed that the signal shadow of the cavernous sinus was less than before, although inflammation was still observed in the right. **e**-**f**: Two months after treatment, MRI showed a normal cavernous sinus and sphenoid sinus

A 34-year-old woman developed a sudden left temporal headache and an ipsilateral toothache. Her toothache was relieved after root canal therapy, but her headache remained unchanged with nonsteroidal anti-inflammatory drugs (NSAIDs) and peripheral nerve blocks. A week later, she developed a completely drooping left eyelid. Then, she was admitted to a Level A tertiary hospital. Her blood routine examination and cerebrospinal fluid (CSF) analysis were normal (Table 1). Brain magnetic resonance imaging (MRI) scan showed abnormal enhancement of the left cavernous sinus (Fig. 1a).

**Table 1:** Laboratory results

**The first headache attack in another hospital on December 11, 2020**			
Test (2020.12.11-2020.12.14)	Results	Reference range	Unit
Blood routine examination			
White blood count	8.77	3.5–9.5	×10^9^/L
Neutrophils	67.2	40–75	%
Blood glucose	5.5	3.9–7.8	mmol/L
Cerebrospinal fluid examination			
Pandy’s test	Negative	Negative	--
CSF cell count	0.003	0.000-0.008	×10^9^/L
CSF WBC count	0.003	0-0.008	×10^9^/L
CSF protein	0.20	0.15–0.45	g/L
CSF glucose	3.55	2.5–4.5	mmol/L
CSF chloride	123.0	120.0-132.0	mmol/L
CSF culture	Negative	Negative	--
**The second headache attack in our hospital on January 10, 2021**			
Test (2021.01.10-2021.01.15)	Results	Reference range	Unit
Blood routine examination			
White blood count	9.47	3.50–9.50	×10^9^/L
Neutrophils	65.4	40.0–75.0	×10^9^/L
C-reactive protein	< 10	0–10	mg/L
Procalcitonin	0.03	0-0.05	ng/ml
Blood glucose	5.8	3.9–7.8	mmol/L
Thyroid Function Tests			
Free triiodothyronine	2.93	2.01–4.82	pg/ml
Free thyronine	0.73	0.59–1.25	ng/dl
Highly sensitive thyroid stimulating hormone	0.810	0.56–5.91	uIU/ML
Antibodies to autoimmune diseases	Negative	Negative	--
Human immunodeficiency virus	0.08	< 1	COI
Cerebrospinal fluid examination			
Pandy’s test	Negative	Negative	--
CSF cell count	0.008	0.000-0.008	×10^9^/L
CSF WBC count	0.002	0.000-0.008	×10^9^/L
CSF protein	0.21	0-0.40	g/L
CSF glucose	3.7	2.5–4.5	mmol/L
CSF chloride	114	111–128	mmol/L
CSF culture	Staphylococcus capitis	Negative	--
Sphenoid sinus puncture fluid culture	coagulase-negative staphylococci	Negative	--

She was given steroid therapy for suspected THS (dexamethasone 15 mg intravenous once daily for 8 days and then oral prednisone 25 mg once daily). Her left blepharoptosis completely resolved, and her headache was completely relieved after 3 days. After 15 days of oral prednisone maintenance therapy, the patient failed to see a doctor for follow-up due to the COVID-19 outbreak and stopped taking medications. However, five days after the discontinuation of steroid treatment, she developed a headache on the right side, so she came to our institution for further evaluation and treatment.

On admission to our hospital, she had a severe throbbing pain in the right temporal region, which was followed by right unilateral blepharoptosis and diplopia and a complete inability to abduct the right eye. A neurological examination showed right abducens nerve palsy, smaller right eye cleft and right pupil, and hypohidrosis of the right hemiface. The patient’s routine blood examination, C-reactive protein, procalcitonin, glycemic curve, and thyroid function tests were normal. Laboratory tests for autoimmune diseases and human immunodeficiency virus (HIV) tests were negative. Serum and CSF antibodies against *Treponema pallidum* and CSF cultures for fungal infection were negative, but a CSF bacterial smear suggested the presence of gram-positive cocci, which were later confirmed to be *Staphylococcus capitis* (Table 1). A subsequent brain MRI showed abnormal soft tissue signals in the bilateral cavernous sinuses, which were more pronounced on the right side, and there were also inflammatory signals in the right sphenoid sinus (Fig. 1b-c). A transsphenoidal approach sphenoid sinus puncture drainage and aspirate culture were completed, and the culture showed the presence of coagulase-negative *staphylococci* (CoNS) (Table 1). Guided by the results of her drug sensitivity test, vancomycin was chosen to be given intravenously for 6 weeks, followed by oral prednisone (started at 30 mg/day and decreased to 10 mg a week) for 4 months. One month after treatment, the patient’s headache completely resolved, and MRI showed a reduction in abnormal signals in the cavernous sinus (Fig. 1d). Two months later, the right ophthalmoplegia had significantly improved, and an MRI showed complete resolution of the inflammation in the bilateral cavernous and sphenoid sinuses (Fig. 1e-f). Three months later, her neurological examination was completely normal.

## Discussion

Cavernous sinus syndrome (CSS) refers to any disease process that affects the cavernous sinus and is characterized by ophthalmoplegia, Horner syndrome, loss of trigeminal nerve sensation, proptosis and edema. Its etiologies include tumor, infection, inflammation, vascular and trauma [10]. THS is a kind of CSS caused by non-specific inflammation of the cavernous sinus area. The main manifestation is painful ophthalmoplegia, and THS significantly responds to systemic steroid therapy [11]. Therefore, it is particularly important to identify this treatable cause and exclude other dangerous causes clinically [12–14].

The estimated incidence of THS is one case per million/year, and it can be unidirectional or can have a relapse-remission course [1]. Half of the patients will have recurrences within a few months or years. These recurrences may be ipsilateral or contralateral, but rarely bilateral [15, 16]. Currently, the diagnosis of THS is based on the improvement of the third edition of the ICHD [9]. In the ICHD-3, the diagnostic criteria for THS require not only the presence of the corresponding clinical manifestations but also a temporal relationship between these manifestations. For example, a patient’s unilateral headache should be accompanied by paresis of the ipsilateral cranial nerves III, IV and/or VI, and the headache should precede the paresis of the cranial nerves by ≤ 2 weeks or develop with it. MRI or biopsy should also be required to confirm the presence of granulomatous inflammation in the symptomatic ipsilateral cavernous sinus, superior orbital fissure or orbit. Most importantly, it is “*not better accounted for by another ICHD-3 diagnosis*” [9]. However, due to the deep location of the cavernous sinus, the abundance of the blood supply, and the complexity of the anatomical region, biopsies are difficult to acquire. Therefore, direct tissue biopsies are rarely performed for diagnosis in actual practice. In addition, when the lesion does not penetrate the dura and enters into the subarachnoid space, CSF samples obtained via lumbar puncture are unlikely to yield positive test results.

Therefore, the clinical diagnosis of THS based on the ICHD-3 diagnostic criterion mainly relies on the combination of typical clinical symptoms and imaging findings. However, there is currently a lack of specific imaging markers that can help distinguish other secondary causes from THS. Research by Chih-Hsien Hung et al. showed that typical symptoms or cranial imaging have a high sensitivity for predicting a diagnosis of THS (95.8% and 100%, respectively) but have a low specificity (47.2% and 28.6%, respectively) [5]. MRI results that are consistent with inflammatory lesions can neither exclude nor confirm THS [17]. Therefore, patients who meet diagnostic criteria A, B and C but do not undergo a biopsy do not fully meet diagnostic criterion D [9]. On the other hand, the timing of MRI may also affect the diagnosis of THS. Radiologically, visible lesions on imaging may take some time to form, suggesting that a normal MRI should not exclude the diagnosis of THS. Previous studies have shown that nearly 50% of THS patients have negative MRI results [18]. These patients cannot be diagnosed according to the current version of the ICHD-3 without evidence from biopsy.

In the previous version of the diagnostic criteria for ICHD, the effect of steroid therapy was also used as one of the diagnostic criteria for THS [19]. Although this criterion was dropped in the third edition, the comments mentioned that remission by appropriate steroid therapy can serve as evidence for considering its diagnosis [9]. Patients who are suspected to have a diagnosis of THS should be treated with steroids. However, patients who are diagnosed with THS without a biopsy are at risk with the use of systemic steroid therapy. Particularly in patients who actually have an infectious cause, steroid therapy may cause their condition to deteriorate rapidly [20]. In our case, the patient’s initial clinical symptoms, MRI manifestations, and response to prednisone were consistent with the diagnostic criteria for THS, so it was understandable to consider THS and give steroid therapy in another hospital, Also, the symptoms were completely relieved after treatment. However, contralateral symptoms developed shortly after discontinuation of prednisone treatment, MRI revealed involvement of the left cavernous sinus area, and the lesion clearly extended beyond the cavernous sinus area. Therefore, failure of treatment prompts us to re-evaluate other possible causes, especially other infections.

The sphenoid sinus is the most overlooked sinus. Compared with that of the other sinuses, the mucosa of the sphenoid sinus wall is pseudostratified ciliated columnar epithelium and contains fewer mucus-secreting cells, so the drainage burden of the sphenoid sinus is lighter. This may be related to the low incidence of sphenoid sinusitis occurring alone [21]. Acute isolated sphenoid sinusitis is rare, accounting for 3% of the incidence of sinusitis [22]. Because of the atypical clinical symptoms and because patients often lack clinical signs, isolated sphenoid sinusitis is commonly missed until the development of neurological symptoms [21]. CoNS account for approximately 50% of the pathogens causing foreign-body-related infections and are often isolated from patients with chronic sinusitis [23, 24]. However, bilateral cavernous sinusitis caused by CoNS is rarely reported. In our case, the patient may have had an infection that originated from her root canal treatment and had spread to her sinuses, or her potential sinus CoNS infection may have been aggravated by her initial steroid therapy. The ICHD-3 entry “11.5 Headache that is attributed to a disorder of the nose or paranasal sinuses” was another potential diagnosis for our patient [9]. Whether acute or chronic, this diagnosis requires headache relief and a progression time that are consistent with changes in sinusitis symptoms. Our patient did not have nasal symptoms throughout the course of the disease, which made it difficult to connect them to the diagnosis at the first visit. This is also one of the reasons for the failure of the first diagnosis.

It has long been recognized that the diagnosis of THS is dangerous and that THS may be clinically overused or misapplied [25]. Some doctors may not strictly adhere to the diagnostic criteria, broadening the definition of THS or equating it with painful ophthalmoplegia or cavernous sinus syndrome due to inflammation. Therefore, it is necessary to standardize the terminology and emphasize etiology to promote the integrity of diagnostic thinking. Some scholars have suggested to not use THS as a diagnostic entity and to merely preserve it as a syndrome without an accurate diagnosis [25, 26]. Furthermore, a recent review from Dutta and Anand recommends dropping the term THS and reverting back to the old terminology of cavernous sinus syndrome (CSS), qualifying it with the terms painful, presumed inflammatory, steroid responsive, recurrent [27]. But in this way, it completely abandons the original definition and understanding of THS. To unify the commonly used terms, we recommend that the ICHD-3 use “cavernous sinus syndrome” (“CSS”) as the umbrella term to cover all syndromes of ophthalmoplegic headache. THS can be classified as an idiopathic inflammatory cause under this category. This can prompt physicians to carry out a thorough investigation according to the etiology of CSS, including vascular, traumatic, neoplastic, infectious and various inflammatory diseases. Labeling a patient as having THS early in the course of disease could be harmful to the patient, especially in patients with reactions to steroids, and this will seriously affect the doctor’s judgment [28]. Using the nomenclature of “CSS” could potentially prevent any presumptive bias regarding the diagnosis and will allow the diagnosis to be modified as the disease progresses.

## Conclusion

This is the first report of cavernous sinusitis caused by coagulase-negative staphylococcal infection. This case suggested that the symptoms of sphenoid sinus infection may mimic THS manifestations. MRI after steroid therapy can be extremely useful and should be performed routinely. Long-term clinical and radiological follow-up is necessary. The use of this cavernous sinus syndrome nomenclature may help to rule out its etiology adequately.

## Data Availability

The data used and analyzed during the present study are available from the corresponding author on reasonable request.

## Abbreviations

THS: Tolosa-Hunt syndrome
ICHD-3: International Classification of Headache Disorders third edition
NSAIDs: Nonsteroidal anti-inflammatory drugs
CSF: Cerebrospinal fluid
MRI: Magnetic resonance imaging
HIV: Human immunodeficiency virus
CoNS: Coagulase-negative staphylococci
CSS: Cavernous sinus syndrome
